# Analyzing conductance correlations involved in recovery of bursting after neuromodulator deprivation in lobster stomatogastric neuron models

**DOI:** 10.1186/1471-2202-13-S1-P37

**Published:** 2012-07-16

**Authors:** Kenneth Shim, Astrid A Prinz, Tomasz G Smolinski

**Affiliations:** 1 Department of Computer and Information Sciences, Delaware State University, Dover, DE 19901, USA; 2 Department of Biology, Emory University, Atlanta, GA 30322, USA

## 

In the crustacean stomatogastric ganglion (STG) functional neuronal activity can be produced with widely varying cellular parameter combinations. This variability has been observed in neurophysiological (*e.g.*, [4]) and computational (*e.g.*, [5]) studies. One possible mechanism responsible for this phenomenon is coregulation of ionic current levels. Although many relationships between ionic currents are cell-specific [6], some appear in several STG neurons. Those include the coregulation of the hyperpolarization-activated inward current, I_h_, and the transient K^+^ current, I_A_, which affects neuronal firing properties [3], and the relationship between the delayed rectifier K^+^ current, I_Kd_, and the transient Ca^2+^ current, I_CaT_, which affects the peak and duration of the slow-wave oscillation in bursting STG neurons [1]. Interestingly, ionic current coregulation depends on neuromodulation, not activity [2]. This is intriguing because the STG can recover function after neuromodulator deprivation (*i.e.*, deafferentation). After the stomatogastric nerve, via which the STG receives neuromodulatory inputs, is cut or blocked, STG neurons initially lose their function. However, within 24h to 96h, without external intervention, they again exhibit activity similar to that in intact STGs. The interplay between deafferentation, function recovery, and coregulation of ionic currents is under active research, which has so far produced interesting results showing that while some relationships are lost due to neuromodulator deprivation, some of them are altered (presumably to support recovery) [8]. Here, we use a computational approach to study these phenomena in an important STG neuron, the anterior burster (AB). Previously, we explored a 12-dimensional parameter space of an AB model by simulating 21,600,000 parameter combinations [7]. Every parameter set was simulated and classified as functional if it produced realistic bursting activity and properly responded to deafferentation (*i.e.*, became quiescent), which was simulated by removal of the modulatory proctolin current. Out of the ~400,000 models that failed the second criterion (*i.e.*, exhibited non-quiescent behavior) we selected those that showed bursting (~14,000). We consider those models “recovered,” as they function despite neuromodulation deprivation. By analyzing the parameter values in the “recovered” neurons, we investigate the impact of deafferentation on coregulations of ionic currents. We show that the relationship between I_h_ and I_A_ is preserved regardless of the presence of neuromodulation, although the slope of the relationship is altered, which coincides with results from two other STG neurons [8]. We also observe the preservation of the I_Kd_ and I_CaT_ relationship, albeit with an altered slope, which has not been reported in AB or any other STG neuron.
